# Essential oil from *Cymbopogon citratus* exhibits “anti-aspergillosis” potential: in-silico molecular docking and in vitro studies

**DOI:** 10.1186/s42269-022-00711-5

**Published:** 2022-01-29

**Authors:** Arun Dev Sharma, Inderjeet Kaur

**Affiliations:** Department of Biotechnology, Lyallpur Khalsa College Jalandhar, Jalandhar, India

**Keywords:** Aspergillosis, COVID-19, Citral oil, Herbal drug

## Abstract

**Background:**

Aspergillosis, has recently confounded some states of India. Due to major role in fungal cell wall synthesis, in the present study UDP-glycosyltransferase, Glucosamine-6-phosphate synthase and chitin synthase were chosen as an appropriate sites to design drug. The objective of present study was molecular docking of lemon grass essential oil component citral and in vitro validation. GC-FID analysis was used to find out aromatic profile. For docking, Patch-dock analysis was used. Ligand Protein 2D and 3D Interactions were also studied. Drug likeliness, and toxicity profile were also studied. Docking analysis indicated effective binding of citral to UDP-glycosyltransferase, Glucosamine-6-phosphate synthase and chitin synthase. In vitro validation was performed by fungal strain *Aspergillus fumigatum.*

**Results:**

GC-FID profiling revealed the presence of citral as major bioactive compound. Interactions results indicated that, UDP-glycosyltransferase, Glucosamine-6-phosphate synthase and chitin synthase enzymes and citral complexes forms hydrogen and hydrophobic interactions. Citral also depicted drug likeliness by LIPINSKY rule, sufficient level of bioactivity, drug likeliness and toxicity.

**Conclusion:**

In vitro results revealed that lemon grass oil was able to inhibit growth of fungal strains toxicity thus signifying its role as potent anti-fungal drug.

**Supplementary Information:**

The online version contains supplementary material available at 10.1186/s42269-022-00711-5.

## Background

The first case of pneumonia caused by SARS-CoV-2 (severe acute respiratory syndrome coronavirus 2) was first reported in China, Wuhan, in December 2019. Afterwards, this viral disease spread rapidly worldwide causing coronavirus disease (COVID-19) a pandemic (Lai et al. 2020). Since the dawn of COVID 19 pandemic, as of August 2020, researchers have documented COVID-19–associated serious co-infections in COVID-19 patients like: aspergillosis, invasive candidiasis, coccidioidomycosis, fusariosis, mucormycosis and saccharomycosis (Alanio et al. 2020; Koehler et al. 2020; Chowdhary et al. 2020; Chang et al. 2020; Poignon et al. 2020). Among all, “aspergillosis” contributed to a high mortality rate of up to 67% (Ventoulis et al. 2020). Aspergillosis is a type of infection which is caused by invasive common mold “Aspergillus”, that exists outdoors and indoors. The rapid rise in fungal infections post 2nd wave of COVID-19 was attributed to the un-regulated use of steroids for COVID patients. It was observed that this fungus is affecting immune-compromised individuals like COVID-19 patients on recovering state and have diabetes or high un-controlled sugar levels (John et al. 2021). It was observed that uncontrolled use of steroids for COVID patients reduced the body immunity and raised blood glucose level in diabetic and non-diabetic individuals due to poor physical activity which increased the rate of infection of fungus infection (Rabagliati et al. 2021). The symptoms of invasive aspergillosis are: running nose, headache, stiffness, chest pain, cough, blood in cough, fever, reduced ability to smell and breathing problems (Schweer et al. 2014).

Due to the rapid emergence of resistant strains of fungus and side effect of antifungal drugs, the synthesis and demand of novel drugs having less toxicity and more effectiveness is instantly required. Hence bioactive compounds that poses properties to act as fungal cell wall-associated enzymes inhibitors have been advocated as key therapeutic drugs to treat fungal infections (Chen et al. 2020). Fungal cell wall is a rigid mechanical barrier that plays a key role in protecting fungus against environmental stresses and other osmotic forces due to the presence of various structural components like chitin, glycosyl phosphatidyl inositol anchors (GPI), glucan and mannoproteins (Han et al. 2017). Therefore, these structural cell wall-based components represent excellent target sites to design antifungal drugs. Chitin synthase, UDP-glycosyltransferase and Glucosamine-6-phosphate synthase are key enzymes involved in cell wall construction. Earlier studies have paved the way that these components can serve as an targets to design antifungal drugs as no such structures exist in human body (Han et al. 2017). We present here our viewpoint that, bioactive molecule citral has the potential to treat fungus infection by targeting fungal enzymes such as: Chitin synthase, UDP-glycosyltransferase and Glucosamine-6-phosphate synthase. Chitin synthase is involved in the process of chitin biosynthesis (Geoghegan et al. 2017). UDP-glycosyltransferase is a key enzyme involved in the first step in glycosyl phosphatidyl inositol GPI biosynthesis. GPI is a potential molecule needed for anchoring proteins to the cell membrane, thus involved in the integrity of the fungal cell wall (Muniz and Zurzolo 2014). Glucosamine-6-phosphate synthase is involved in the syntheses of N-acetylglucosamine which is an essential building block for fungal cell wall chitin (Banerjee et al. 2011).

Lemon grass essential oil (LGO) from *Cymbopogon* species, also known as lemon grass, encompasses a number of bioactives. Due to the complex nature of essential oil, their anti-fungal mechanism of action is still not completely understood (Elaissi et al. 2011). LGO has been used as complementary and traditional medicine in ancient times.

This study postulated that due to the richness of citral, essential oil from *Cymbopogon citratus* plants have the potential to inhibit “Aspergillosis”. Hence as an objective this study was designed to study molecular docking of citral against Chitin synthase, UDP-glycosyltransferase and Glucosamine-6-phosphate synthase and wet-lab validation. Various potent biological activities like anti-amoebic, anti-inflammatory, anti-filarial, anti-diarrheal, anti-malarial, and anti-bacterial agent, anti-HIV have been attributed to LGO, hence playing a major role as therapeutics in the scientific community (Oladeji et al. 2019; Shah et al. 2011). However, its role against “Aspergillosis” is still not well documented. Hence, the novelty of present study lies in the fact that by using LGO due to having richness of bioactive citral and other minor bioactives as new anti- Aspergillosis drug. It would further offer new insights into the potential prospects to identify the key antifungal drugs during COVID19 medications.

## Methods

### Gas chromatography analysis

Gas chromatography (GC-FID) analysis was performed using a Chemtron 2045 gas chromatograph coupled with FID (flame ionization detector). A 2 m long column of stainless steel filed with 10% OV-17 on 80–100% mesh Chromosorb W (HP) was used. Carrier gas was Nitrogen at flow rate of 30 ml/min. The detector and injector temperatures were kept at 210 °C and 260 °C and 0.1 µl sample was injected. Ramping conditions for oven were: 110 °C (initially maintained) ramped to 200 °C at 2 °C/min. Bioactive molecules were identified by comparison of their relative retention times with either those of known standards or with published data in the literature and matching their mass GC-FID spectra with the NIST spectral libraries spectra (Adams 2012).

### Ligand modelling

Citral was ligand for Chitin synthase, UDP-glycosyltransferase and Glucosamine-6-phosphate synthase structures. SMILES of citral was retrieved from NCBI-Pubchem database for molecule. UCSF-chimera was used to build 3D structure.

### Protein receptor preparation and molecular docking

X-ray crystal structures of Chitin synthase, UDP-glycosyltransferase and Glucosamine-6-phosphate synthase were retrieved from PDB web cite (https://www.rcsb.org/). The target enzymes were cleaned from co-crystallized ligand, selected water molecules and cofactors, prepared energy minimized before docking study. Before the docking studies, the protein structure was first prepared using the dock prep set up in chimera software. The dock preparation is an optimization part that corrects atomic and bond length, structure, charges anomalies. Original inhibitors and water molecules were detached from the Chitin synthase, UDP-glycosyltransferase and Glucosamine-6-phosphate synthase structures and any missing hydrogen atoms were added. PatchDock tool was used for docking study of the citral over Chitin synthase, UDP-glycosyltransferase and Glucosamine-6-phosphate synthase enzyme (https://bioinfo3d.cs.tau.ac.il/PatchDock/). For this both ligand (citral) and receptors molecules in.pdb file formats were uploaded to the PatchDock server and docking was executed. The best generated docked structure was downloaded and saved as.pdb file. Biovia Discovery Studio Visualizer 2020, Chimera tools and Plip tool were used to study docked complexes and their 2D and 3D interactions.

### Drug-likeness and toxicity

Citral was retrieved from PubChem (https://pubchem.ncbi.nlm.nih.gov/) with PubChem CID16142. Physiochemical properties, drug likeness and pharmacokinetics studies and ADMET (Absorption, Metabolism, Toxicity and Excretion) were conducted using SWISSADME (http://www.swissadme.ch/http://lmmd.ecust.edu.cn/admetsar1/predict/). Toxicity profile was studied by using ProTox-II webserver (http://tox.charite.de/protox_II). It calculates prediction based on different levels of toxicity such as organ toxicity (hepatotoxicity), oral toxicity, toxicological endpoints (such as cytotoxicity, carcinotoxicity, mutagenicity and immunotoxicity). Web based molinspiration tool was used to evaluate the bioactivity potential of citral (https://www.molinspiration.com/cgi-bin/properties).

### Active sites prediction in 3D modeled receptor

CASTp (The Computed Atlas of Surface Topography of proteins) web tool was used to predict active sites in the Chitin synthase, UDP-glycosyltransferase and Glucosamine-6-phosphate synthase proteins. CASTp is an online tool used in identification and dimension of cavities on 3D protein structures. Default value of 1.4 Angstroms was used as probe radius.

### In-vitro antifungal activity of lemon grass oil

Fungal cultures; *Aspergillus fumigatum* (MTCC 343) was procured from Institute of Microbial Technology, Chandigarh (India). Strains were maintained on Potato Dextrose Agar media (Hi-media). Leaves of *Cymbopogon citratus* was collected from campus fields and plots. Lemon grass oil from leaves was extracted by using Steam-distillation method as described by Agnish et al. (2021). Oil obtained was stored in dark bottles at 4 °C till further use. The antifungal activity of LGO was determined according to poison food technique. In this method, 20 mL of PDA media and different concentration of LGO (50–100 μλ/ml) were prepared. Agar discs with mycelia (10 mm diameter) were cut from periphery of actively growing region of 7-day old pure culture of *Aspergillum fumigatum* by using a sterile cork borer and aseptically inoculated in centres of PDA agar plates. Negative control (Blank) plates without LGO were also made. 250 μλ of 50 × streptomycin solution (0.5% in 0.9%PBS) was used as positive control. All plates were incubated at 37 °C for 7 days.

### Determination of minimum inhibitory concentration (MIC) of essential oil

MIC of LGO against *Aspergillum fumigatum* was determined according to zone of inhibition agar disc diffusion method as described by Agnish et al. (2021) with some modifications. MIC was performed at six different concentrations of the essential oil (25–100 µl). Briefly, 10 agar discs with mycelia (10 mm in diameter) were taken from periphery of actively growing 7-day old pure culture of *Aspergillum fumigatum* suspended in 15 mL of sterile H_2_O. 100 μλ of this fungal suspension was used as inoculums and was evenly spread on petri plates containing PDA media. The plates were kept standing at room temperature for 20 min. Sterilized paper disc (15 mm in diameter) impregnated with different aliquot of LGO were placed in centre of each agar plates and incubated at 37 °C for 7 days. The MIC was defined as the lowest concentration of an essential oil that inhibited the visible growth of a fungus after overnight incubation.

## Results

### GC-FID analysis of bioactive molecules in LGO

**Fig. 1:**
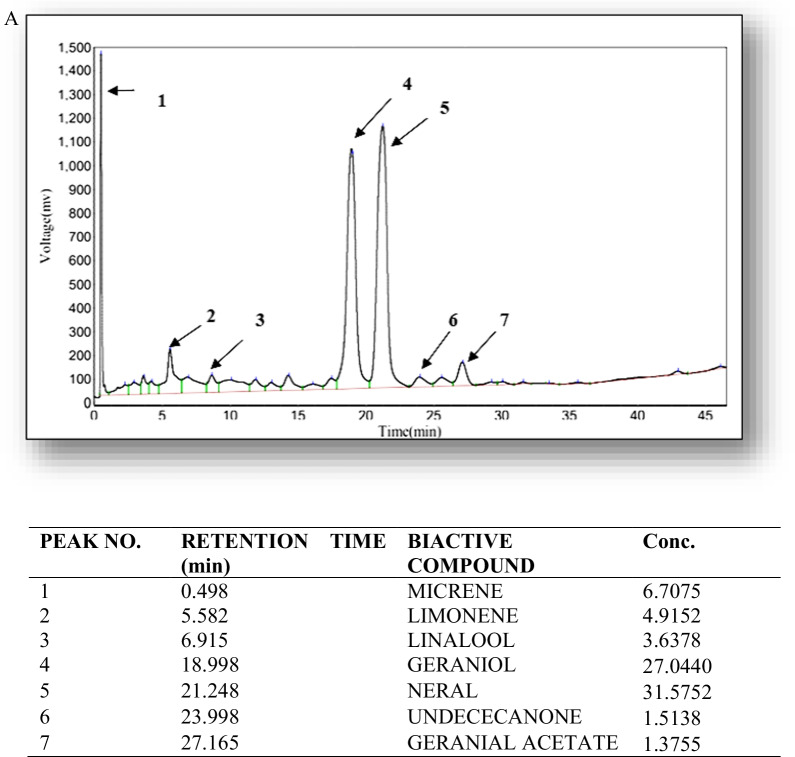

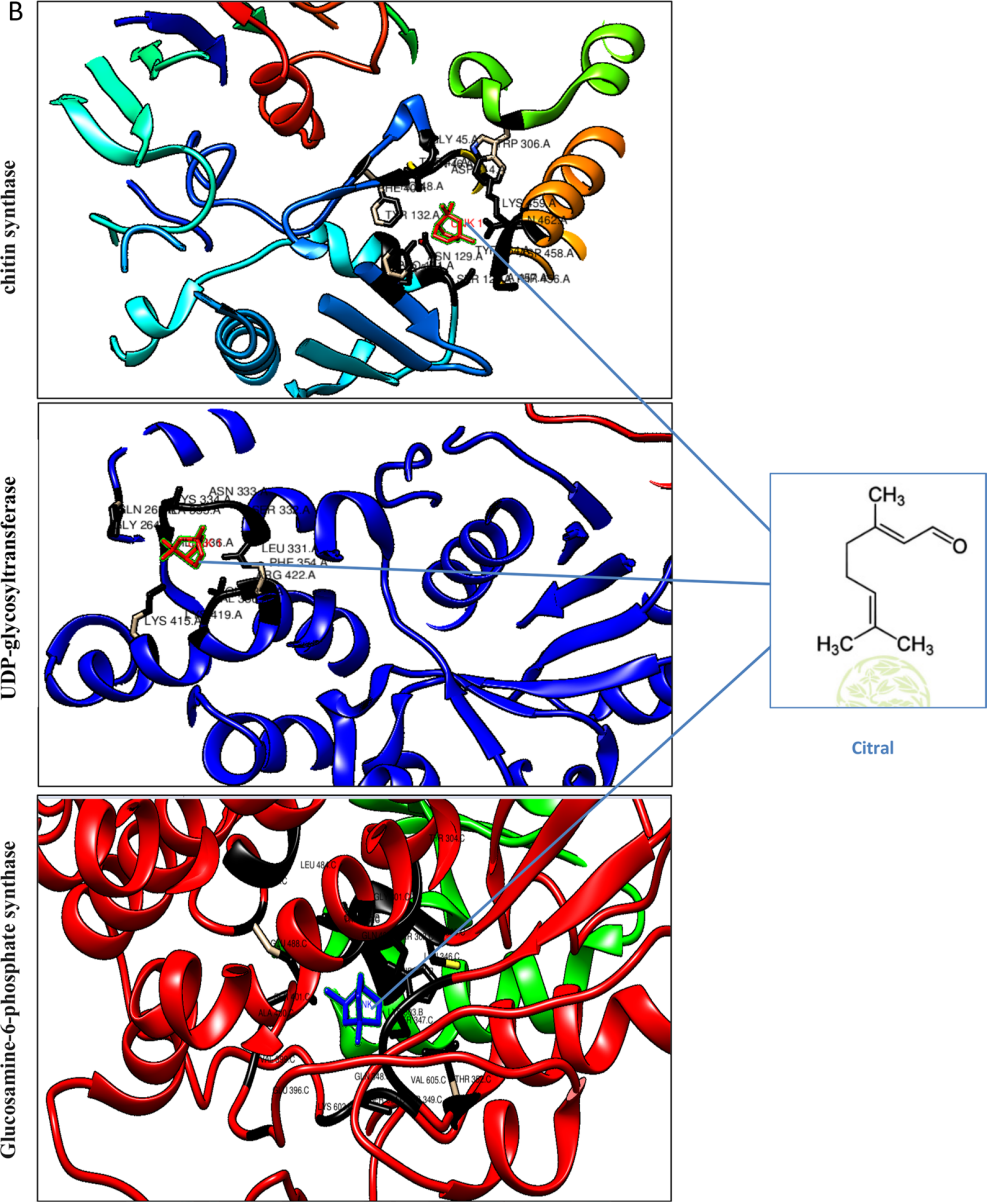
**A** GC-FID analysis of Lemongrass essential oil. **B** Molecular docking of citral with fungal cell wall receptors

The GC-FID chromatogram obtained was depicted in Fig. 1a. The peaks observed and their respective retention time was also displayed. The GC- FID analysis of lemon grass oil obtained from *Cymbopogon citratus* revealed 26 compounds for the total of 100%. In the present study, all identified compounds were micrene, limonene, linalool, geraniol, neral, undececanone and geranial acetate. GC-FID chromatogram contained three major peaks along with many small peaks indicating the presence of major compounds. The major constituents were geraniol (27%, Citral-a), neral (31%, Citral-b). The small peaks may be ascribed to the disintegrated major bioactive compounds present in small quantities.

### Molecular docking

This study investigated docking of citral bioactive molecule from lemon grass oil as key fungal inhibitor candidates against chitin synthase, UDP-glycosyltransferase and Glucosamine-6-phosphate synthase enzymes. *In-silico* docking results based on dock score and area are demonstrated in Table 1. Among all enzymes, it was found that Glucosamine-6-phosphate synthase depicted strong docking with citral as evident from dock score of 3778. Dock core for chitin synthase and UDP-glycosyltransferase was 3288 and 3416, respectively. Docking pose and molecular interactions of citral with chitin synthase, UDP-glycosyltransferase and Glucosamine-6-phosphate synthase are shown in Fig. 1b. It was observed that citral successfully docked in the active sites of chitin synthase, UDP-glycosyltransferase and Glucosamine-6-phosphate synthase.

**Table 1:** Molecular docking of fungal receptors with citral

Fungal receptor	Dock score				Interacting residues within 4 A˚ radius		
	Score	Area	ACE	Transformation	Water bridge	Hydrophobic interactions	Hydrogen bonds
Chitin synthase	3288	347.80	− 77.93	− 0.04 0.77 − 2.94 − 5.19 − 29.99 14.59	–	TYR25, PRO177	–
UDP-glucosyltransferase	3416	410.80	− 78.29	3.13 − 0.12 0.57 66.51 76.17 60.31	MET58	PHE40, ALA457	–
Glucosamine-6-phosphate synthase	3778	421.90	− 68.28	2.08 0.98 − 0.27 3.02 26.91 80.05	–	LY35, THR37	SER101, ASN 103

**Fig. 2:**
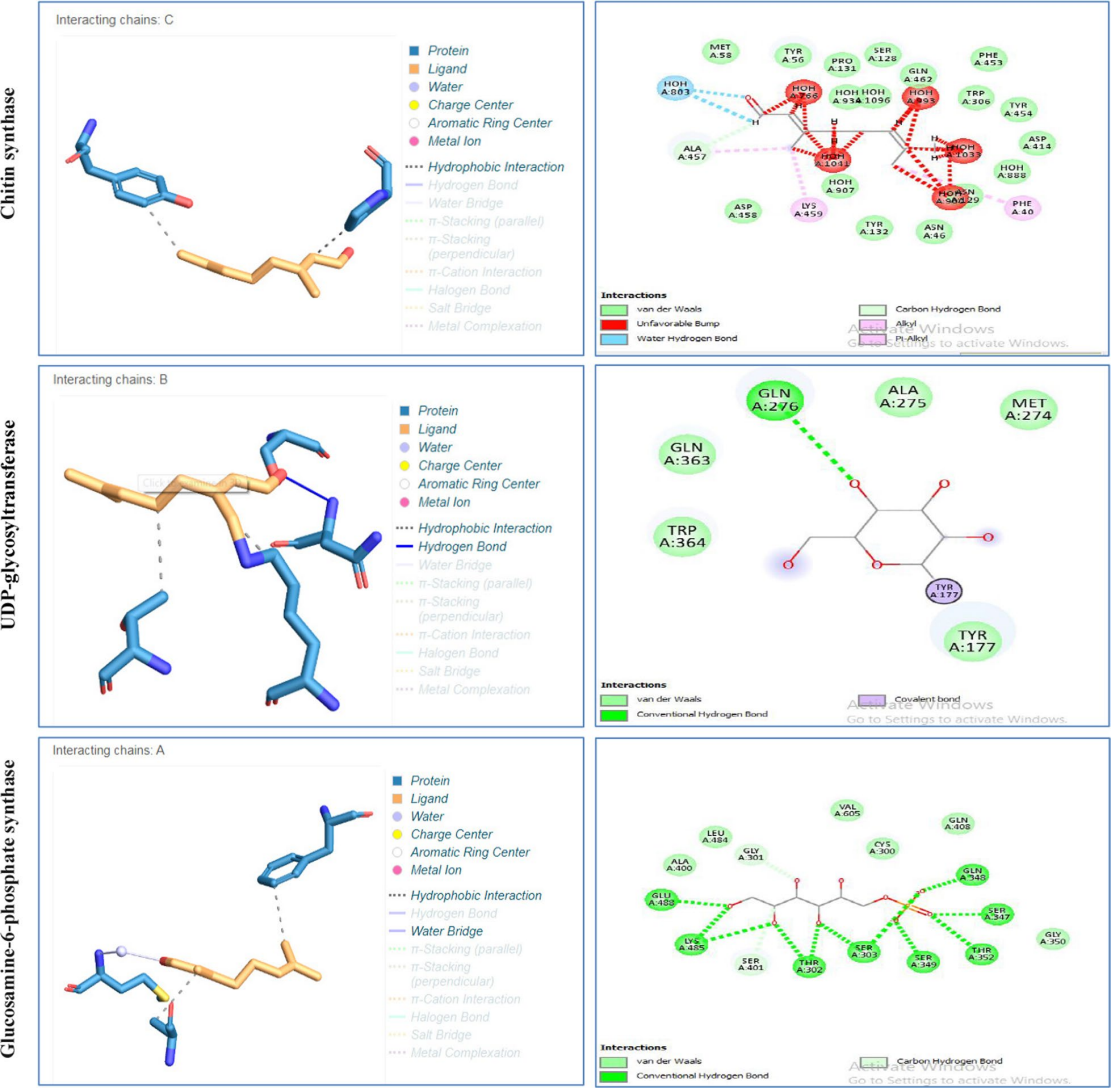
2D and 3D interactions of Citral with protein receptors

During docking, drug molecule either forms hydrophobic interactions or hydrogen bonding with the active site residues of receptor that determines affinity of ligand with receptor. Molecular interactions of citral with chitin synthase, UDP-glycosyltransferase and Glucosamine-6-phosphate synthase were further evaluated. It was observed that the interaction of citral in active sites of chitin synthase, UDP-glycosyltransferase and Glucosamine-6-phosphate synthase were mediated by both hydrophobic and hydrogen bond interactions. With Glucosamine-6-phosphate synthase hydrophobic interactions were observed via LYS 35 and THR 37 (Fig. 2). Hydrogen bond interactions of citral with Glucosamine-6-phosphate synthase were also observed via SER 101 and ASN 103. For chitin synthase, hydrophobic interactions were observed via PRO103, TYR 25. With UDP-glucosyltransferase, hydrophobic interactions were observed via PHE40 and ALA457. No Hydrogen bond interactions were observed with chitin synthase, UDP-glycosyltransferase. Active site prediction by CAST-P server indicated interacting residues in the major cavity of chitin synthase, UDP-glycosyltransferase and Glucosamine-6-phosphate synthase enzymes (Table 2). With CASTp, a major pocket was identified with Area (SA) of 939 and Volume (SA) of 3369 in chitin synthase. While Area (SA) of 1648 and Volume (SA) of 1198 were observed for Glucosamine-6-phosphate synthase.

**Table 2:** Protein target structure, native ligand and active site amino acids

Pdb id	Macromolecule	Native ligand	Interacting active site residues	Cavity	
				Area	Volume
4gf8			TYR 454, ASP458, THR456,457, PRO48, 131, LYS459, ASN129, PHE40, SER128, TYR 132,454, TRP306, ALA457, GLN412	939.053	3369.091
5u6m			ASN333, 332, LEU 331, PHE354, LYS415, 419, 334, ARG422, SER332, ALA335,VAL 356	1087.766	1096.959
1jxa			ALA400, VAL399, GLY 505, HIS 504, TYR 304, GLN348, SER 303, LEU 346, LYS 603, GLU 396, GLN 348, CYS300, THR352, 302, GLU 396	1648.961	1198.342

### PASS analysis, *in-silico* bioactivity, cell toxicity and ADMET properties

**Fig. 3:**
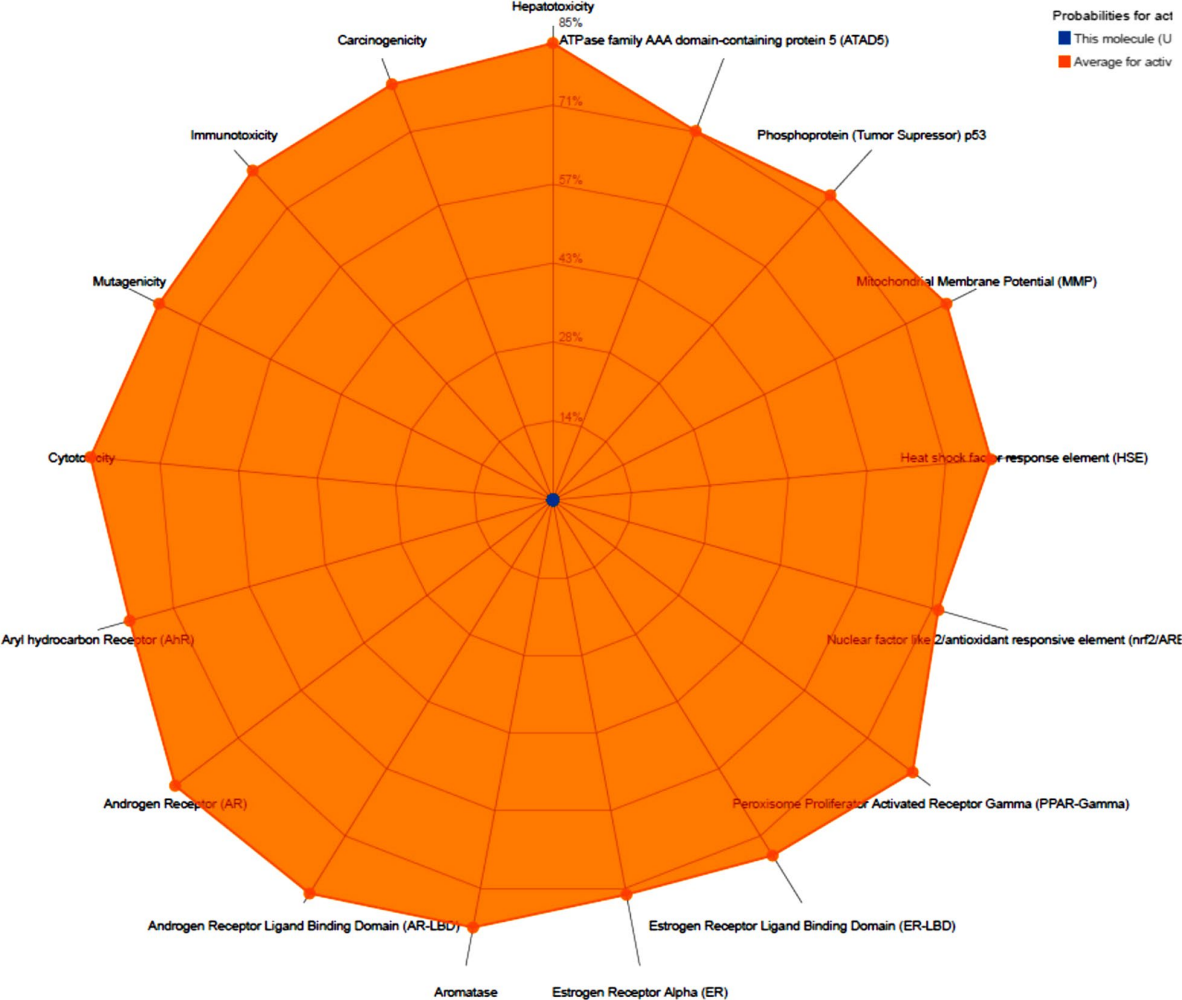
Toxicity radar chart of citral, toxicity profile of the input compound is shown using orange dots/lines which represents the predicted probabilities of the input compound for respective ProTox-II models

To find out drug likeliness, Lipinski rule of 5 (RO5) is generally used. It is based on some molecular parameters like TPSA (polar surface area), mlog P (partition coefficient), number of hydrogen bond donors, molecular weight and number of hydrogen bond acceptors. According to this rule for drug like properties ligands should have log P ≤ 5, number of H-bond acceptors ≤ 10, and H-bond donors ≤ 5 and no more than 1 violation. As shown in Table 3, citral has shown good agreement with the given criteria. *In-silico* absorption of citral was 100%. It was observed that citral was low molecular weight ligand. The Log *P*_o/w_ value was also in acceptable range. The surface view depicting molecular lipophilicity potential (MLP) is also shown in Fig. 3. Topological polar surface area (TPSA) value was 17. 07 Å squared.

**Table 3:** ADME properties of citral

Physicochemical properties	
Molecular weight	152.23 g/mol
Num. heavy atoms	11
Num. arom. heavy atoms	0
Fraction Csp3	0.50
Num. rotatable bonds	4
Num. H-bond acceptors	1
Num. H-bond donors	0
Molar refractivity	49.44
TPSA?	17.07 Å^2^

Lipophilicity	
Log *P*_o/w_ (iLOGP)?	2.47
Log *P*_o/w_ (XLOGP3)?	3.03
Log *P*_o/w_ (WLOGP)?	2.88
Log *P*_o/w_ (MLOGP)?	2.49
Log *P*_o/w_ (SILICOS-IT)?	2.65
Consensus Log *P*_o/w_?	2.71

Water solubility	
Log *S* (ESOL)?	− 2.43
Solubility	5.67e−01 mg/ml; 3.73e−03 mol/l
Class?	Soluble
Log *S* (Ali)?	− 3.05
Solubility	1.34e−01 mg/ml; 8.83e−04 mol/l
Class?	Soluble
Log *S* (SILICOS-IT)?	− 1.96
Solubility	1.66e+00 mg/ml; 1.09e−02 mol/l
Class?	Soluble

Pharmacokinetics	
GI absorption?	High
BBB permeant?	Yes
P-gp substrate?	No
CYP1A2 inhibitor?	No
CYP2C19 inhibitor?	No
CYP2C9 inhibitor?	No
CYP2D6 inhibitor?	No
CYP3A4 inhibitor?	No
Log *K*_p_ (skin permeation)?	− 5.08 cm/s

Druglikeness	
Lipinski?	Yes; 0 violation
Ghose?	No; 1 violation: MW < 160
Veber?	Yes
Egan?	Yes
Muegge?	No; 2 violations: MW < 200, Heteroatoms < 2
Bioavailability score?	0.55

Medicinal chemistry	
PAINS?	0 alert
Brenk?	3 alerts: aldehyde, isolated_alkene, michael_acceptor_1?
Leadlikeness?	No; 1 violation: MW < 250
Synthetic accessibility?	2.49

Bioactivity was calculated with online Molinspiration software based on following parameters like: GPCR ligand, Ion channel modulator,Kinase inhibitor, Nuclear receptor ligand, Protease inhibitor and Enzyme inhibitor. This score as per rule is calculated in three different ranges: score > 0, drug is active, if it is between -5.0 and 0, drug is judiciously active and if score < than − 5.0, drug is quiet. For citral, mostly bioactivity score was in the range of − 5.0 and 0, indicated citral to be act a potential drug (Table 4). Toxicity profile revealed that citral bioactive molecule was mostly non-toxic to organs as inactive prediction was observed like hepatotoxicity (Table 5). Further, citral was non-carcinogenic and non-mutagenic in nature. Toxicity radar chart is also shown in Fig. 3, that quickly exemplifies the assurance of positive toxicity outcomes compared to the average of its class. The data displayed is the average probability of its active class, acquired by computing from the training set data for each model. The prediction probability of citral for all models was lower than the average probability of the training set active compounds thus depicting inactive.

**Table 4:** Bioactivity score of citral

Bioactivity	Score
GPCR ligand	− 0.86
Ion channel modulator	− 0.25
Kinase inhibitor	− 1.29
Nuclear receptor ligand	− 0.42
Protease inhibitor	− 0.57
Enzyme inhibitor	0.02

**Table 5:** Toxicity profile of citral

Classification	Target	Prediction
Toxicity end points	Carcinogenicity	Inactive
Toxicity end points	Mutagenicity	Inactive
Toxicity end points	Cytotoxicity	Inactive
Tox21-nuclear receptor signalling pathways	Aryl hydrocarbon Receptor (AhR)	Inactive
Tox21-nuclear receptor signalling pathways	Androgen Receptor (AR)	Inactive
Tox21-nuclear receptor signalling pathways	Androgen Receptor Ligand Binding Domain (AR-LBD)	Inactive
Tox21-nuclear receptor signalling pathways	Peroxisome Proliferator Activated Receptor Gamma (PPAR-Gamma)	Inactive
Tox21-stress response pathways	Nuclear factor (erythroid-derived 2)-like 2/antioxidant responsive element (nrf2/ARE)	Inactive
Tox21-stress response pathways	Heat shock factor response element (HSE)	Inactive
Tox21-stress response pathways	Mitochondrial Membrane Potential (MMP)	Inactive
Tox21-stress response pathways	Phosphoprotein (Tumor Supressor) p53	Inactive
Tox21-stress response pathways	ATPase family AAA domain-containing protein 5 (ATAD5)	Inactive

### In vitro antifungal activity of lemon grass oil

**Fig. 4:**
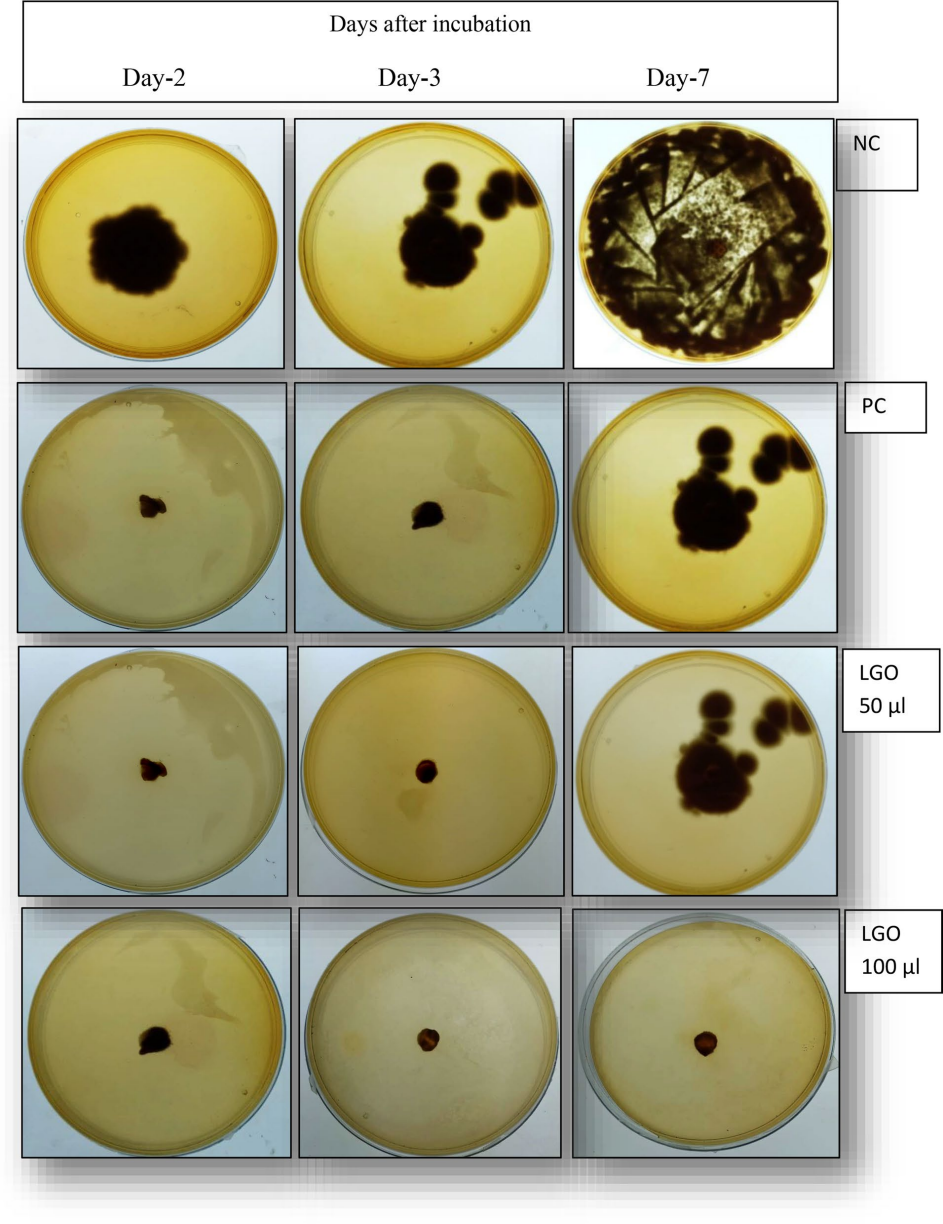
Anti-fungal activity of Lemon grass oil (LGO) against *Aspergillum fumigatum*. NC: negative control. PC: positive control

In order to validate the *in-silico* findings, wet-lab experiment was designed to evaluate antifungal potential of lemon grass oil against fungal strains: *Aspergillus fumigatum*. In fungal strain, at 50 µl essential oil concentration during 2 to 3 days, no fungal growth was observed. However, some fungal growth was observed after 7 days of incubation. On the contrary, at 100 µl concentration, complete mycelial growth inhibition was observed during 2 to 7 days of incubation (Fig. 4, Additional file 1: Fig. S1). Negative control plates depicted sufficient fungal growth even at 2 and 3 days of incubation. Full lawn was observed after 7 days of incubation. Positive plates did not shown any sign of fungal growth at 2 and 3 days but shown some fungal growth at 7 days of incubation. MIC studies of LGO were also conducted using six different concentration of lemon grass essential oil. It was observed that at lower concentrations (15–35 µl), some marked inhibition in fungal growth was observed. However, no growth was observed at 100 µl of LGO (Additional file 1: Fig. S1).

## Discussion

Earlier observations cited that COVID patients who are in immune-compromised condition or having uncontrolled diabetes are infected by “Aspergillosis”. Studies revealed that drugs targeting fungal cell wall components like chitin synthase, UDP-glycosyltransferase and Glucosamine-6-phosphate synthase can be promising antifungal therapeutic agents as no such structure exits in humans (Gong et al. 2017; Han et al. 2017). Hence, chitin synthase, UDP-glycosyltransferase and Glucosamine-6-phosphate synthase may offer new active fungicidal approach to treat Aspergillosis. Our GC-FID phytochemical based studies revealed that citral is a main bioactive of lemon grass oil in *Cymbopogon citratus*.

### GC-FID analysis of bioactive molecules in LGO

The GC- FID chromatogram obtained was depicted in Fig. 1a. The peaks observed and their respective retention time was also displayed. The GC- FID analysis of lemon grass oil obtained from *Cymbopogon citratus* revealed 26 peaks for the total of 100%. In the present study, seven compounds were identified such as: micrene, limonene, linalool, geraniol, neral, undececanone and geranial acetate. GC-FID chromatogram contained major peaks along with many small peaks indicating the presence of other minor bioactive compounds**.** The major constituent was citral which comprised of a mixture of two terpenoids geometric isomers, neral (31%, Citral-a, *E*-isomer) and geranial (27%, Citral-a, *Z*-isomer) (Pihlasalo et al. 2007). Earlier studies also documented citral as a major constituent in the lemon grass oil from other varieties of *Cymbopogon* (Rao et al. 2015; Hanaa et al. 2012; Ganjewala, and Luthra 2010; Ganjewala 2009). Ganjewala (2009) also reported antimicrobial, antiparasitic, antispasmodic, analgesic, anti-inflammatory activities of citral. Due to its distinct, acceptable, and passionate lemon-like pleasant odor, it is natural additive used in foods beverages, and cosmetics (Zeng et al. 2015). *Cymbopogon citratus* essential oils have been established to show antimicrobial, antifungal, and anti-parasitic properties (Zeng et al. 2015).

### Molecular docking

Among all, structure-based drug design (SBDD) is most commonly used, which is based on 3-D structure (Singh et al. 2016a, b). In SBDD, Molecular docking is a key technique that can be applied in designing drug making process. *In-silico* docking has facilitated researchers to monitor conformations and affinities of a collection of bioactive compounds against receptors (Barcellos et al. 2019). This study investigated the docking of citral bioactive molecule from lemon grass oil as key fungal inhibitor candidate against chitin synthase, UDP-glycosyltransferase and Glucosamine-6-phosphate synthase enzymes. It was observed that citral successfully docked in the active sites of with chitin synthase, UDP-glycosyltransferase and Glucosamine-6-phosphate synthase. Chitin synthase is a membrane bound enzyme complex having three domains: an N-terminal domain, a catalytic domain and a C-terminal transmembrane domain. From *in-silico* analysis it was found that citral exhibited its interaction with catalytic domain involved in chitin chain elongation between N and C terminal domains (Dorfmueller et al. 2014). Glucosamine-6-phosphate synthase posse N-terminal and C-terminal ones, catalyzing glutamine hydrolysis and sugar-phosphate isomerization (Mouilleron et al. 2008; Wojciechowski et al. 2005). Computational analysis revealed that citral successfully docked with C terminal domain involved in sugar-phosphate isomerization. UDP-glycosyltransferase enzyme has two-domain structures: N-domain for binding site of the aglycone substrate and C- terminal likely site of UDP-glucose binding (Sawitri et al. 2018). Docking analysis exhibited interaction of citral with C –terminal UDP-glucose binding domain. These results are in agreement with those of (Omar et al. 2021; Biswal et al. 2019) as they stated that molecular docking analyses were performed to clarify the antifungal effectiveness of the most active compounds of essential oil from *Trachyaspermum ammi, Thymus vulgaris* and *Boswellia carteri* against fungal enzymes. This study indicates that eucalyptus essential oil may be considered as the most important sources of antifungal compounds.

During docking, drug molecule either forms hydrophobic interactions or hydrogen bonding with the active site residues of receptor that determines affinity of ligand with receptor. So molecular interactions of citral with chitin synthase, UDP-glycosyltransferase and Glucosamine-6-phosphate synthase were further evaluated. It was observed that the interaction of citral in active sites of chitin synthase, UDP-glycosyltransferase and Glucosamine-6-phosphate synthase were mediated by both hydrophobic and hydrogen bond interactions. Greater the hydrogen bonds between the enzyme and ligand determines the strength of binding (Kortemme et al. 2003). In view of this, due to having hydrogen bond interaction, citral depicted strong binding with Glucosamine-6-phosphate synthase as compared to other enzymes which was also evident from its docking score. Active site prediction by CAST-P server indicated interacting residues in the major cavity of chitin synthase, UDP-glycosyltransferase and Glucosamine-6-phosphate synthase enzymes. Since citral poses high affinity towards chitin synthase, UDP-glycosyltransferase and Glucosamine-6-phosphate enzymes so it was postulated that chitin synthase, UDP-glycosyltransferase and Glucosamine-6-phosphate proteins becomes closed upon binding with citral that in turn induces conformational change in chitin synthase, UDP-glycosyltransferase and Glucosamine-6-phosphate proteins and stop further execution of catalysis of fungal cell wall synthesis hence down regulate the infectivity and Aspergillus into the host cell. The present study results were in consonance with the earlier *in-silico* findings suggesting that polypharmacological agents via cell wall inhibition can act as a therapeutic for the management of Aspergillosis among COVID-19 patients (Lima et al. 2019; Geoghegan et al. 2017).

### PASS analysis, *in-silico* bioactivity, cell toxicity and ADMET properties

For therapeutic use of drugs in living organisms, ADMET properties (absorption, distribution, metabolism, excretion and toxicity) are very imperative for the success of any drug (Wu et al. 2020). To find out drug likeliness, the Lipinski rule of 5 (RO5) is generally used. It is based on some molecular parameters like TPSA (polar surface area), mlog P (partition coefficient), number of hydrogen bond donors, molecular weight and number of hydrogen bond acceptors. According to this rule for drug-like properties ligands should have log P ≤ 5, number of H-bond acceptors ≤ 10, and H-bond donors ≤ 5 and no more than 1 violation. As shown in Table 3, citral has shown good agreement with the given criteria. Hence, it was postulated that bioactive compound citral could be considered an oral drug (Biswal et al. 2019). *In-silico* absorption of ciral was 100%. It was observed that citral was a low molecular weight ligand. It was cited that low MWT compounds are easily diffused and transported across the biological membranes as compared to high MWT compounds (Srimai et al. 2013). The Log *P*_o/w_ value was also in acceptable range. In rational drug design and pharmacokinetic analysis, Log *P*_o/w_ is a key parameter to assess the lipophilicity of any drug and its distribution in body after absorption (Abraham 2003). Topological polar surface area (TPSA) value was 17. 07 Å squared. Topological polar surface area is a key predictor of drug transport properties such as nice permeability, bioavailability and intestinal absorption (Wu et al. 2020). GI (Gastrointestinal tract absorption) of citral was high (Table 3). In order to exert a toxic effect, drug molecules have to be absorbed from intestinal tract in the body. Further, citral was non substrate to efflux transporters such as P-glycoprotein (P-gp). In the gut, P-glycoprotein pumps drugs back into the lumen, decreasing their absorption (Konig and Muller 2013). Citral bioactive compound elaborated non-inhibitory potential against CYP450 series of enzymes, involved in liver detoxification in body (Srimai et al. 2013; Abraham 2003). These observations indicated that citral can easily interact with target receptors and can be further taken in the evaluation of biological activity score.

Biological activity is a key parameter which describes the effect of a drug in living systems. In living systems, ligands have to be bound to biological targets which are also known as drug targets (Khan et al. 2017). Drug targets mostly include common proteins such as enzymes, receptors and ion channels. Bioactivity score was calculated with online Molinspiration software based on following parameters such as binding to GPCR ligand, Ion channel modulator, Kinase inhibitor, Nuclear receptor ligand, Protease inhibitor and Enzyme inhibitor. This score as per rule is calculated in three different ranges: score > 0, drug is active, if it is between − 5.0 and 0, drug is judiciously active and if score < than − 5.0, drug is quiet. For citral, bioactivity score for Ion channel modulator was 0.01 whereas for GPCR ligand, Kinase inhibitor, Nuclear receptor ligand, Protease inhibitor and Enzyme inhibitor score was in the range of − 5.0 and 0, (Table 4). All these observations indicated that citral possess such properties as are required for the bioactive molecules to act as potential drugs. Similar observations have been reported by researchers working on different drug formulations (Khan et al. 2017; Dar et al. 2016). The bioactivity score deliver the evidence about the binding cascade of the citral that is used for the improvement of a new functional drug with increased binding selectivity profile and less undesirable effects (Khan et al. 2017).

For pharmaceutical industries, proper risk assent of a chemical drug is a prerequisite to assess the safety profile of a therapeutic drug (Banerjee et al. 2018). In this regard, in silico toxicity is a key platform to evaluate toxicity prediction of drugs that could be detrimental to humans, animals, and environments (Raies and Bajic 2016). Thus toxicity profile of citral was evaluated and toxicity profile revealed that citral bioactive molecule was mostly non-toxic to organs as inactive prediction was observed like hepatotoxicity (Table 5). Drug-induced hepatotoxicity is the major reason for the liver damage and main reason for the un-success of major drugs in the market (Siramshetty et al. 2016). Further, citral was non-carcinogenic and non-mutagenic in nature. Mutagenic nature of biomolecules is harmful to cell and is the main reason behind certain diseases, e.g. cancer (Lea et al. 2017). Further citral showed inactiveness towards targets based biological pathways like Nuclear receptor signaling pathways and Stress response pathways. All these targets like aryl hydrogen receptor (AhR), androgen receptor (AR), androgen receptor ligand binding domain (AR-LBD), 2/antioxidant responsive element (ARE), heat shock factor response element (HSE), mitochondrial membrane potential (MMP) are important components of biological system inside human body (Huang et al. 2016). Toxicity radar chart is also shown in Fig. 3, that quickly exemplify the assurance of positive toxicity outcomes compared to the average of its class.

### In vitro antifungal activity of lemon grass oil

In order to validate the *in-silico* findings, wet-lab experiment was designed to evaluate antifungal potential of lemon grass oil against fungal strains: *Aspergillus fumigatum*. In fungal strain, although some fungal growth was observed using 50 µl essential oil concentration after 7 days of incubation, however, complete mycelial growth inhibition was observed at essential oil concentration of 100 µl (Fig. 3) after 7 days of incubation. The strong antifungal activity of lemon grass essential oil may be due to the richness of bioactive compounds. These results were supported by the work of Boukhatem et al. (2013) and Tavares et al. (2014) citing inhibitor role of lemon grass role against athlete's foot, ringworm, jock itch and yeast infections. These authors cited that the possible reason behind high antifungal activity of LGO was its high citral and polyphenolic content. Citral, a monoterpene occurs naturally in herbs, plants and citrus fruits and possesses high antifungal, insecticidal and bactericidal activity. Due to the high antifungal activity of LGO, it is used in nasal sprays for prevention of fungal infections in nose and respiratory tract (Ortiz et al. 2010). The possible anti-fungal mechanism is that due to lipophilicity nature of LGO, it easily penetrates fungus cell wall that finally inhibits fungal hyphal-growth or a decrease in the production and germination of conidia of fungal strains. Gao et al. (2016) also reported that at a high concentration of oils, the fungal hyphae collapse and break down which finally damages the cytoplasmic membrane, leading to the leakage of electrolytes and possibly lipid peroxidation due to ROS generation induced by the increase in permeability. Singh et al. (2016a, b) also demonstrated that due to the presence of bioactive like geraniol, that is found abundantly in lemongrass and aromatic herbs, essential oils may induce an inhibitory effect on the calcineurin pathway which leads to a decrease in the plasma membrane permeability and damage of the cell wall.

## Conclusions

Aspergillosis has emerged as a pandemic worldwide. The findings emanated from docking studies revealed that lemon grass essential oil due to the richness of citral could be promising antifungal therapeutic agents against chitin synthase, UDP-glycosyltransferase and Glucosamine-6-phosphate synthase protein. This finding as further validated by *in-vitro* wet lab experiments against fungal strain *Aspergillus fumigatum* where complete mycelial growth inhibition was observed. All these findings could be used as promising data to design and synthesize antifungal drugs during COVID-19 medication by pharmaceutical and medicine based companies.

## Supplementary Information

**Additional file 1: Supplementary Figure 1**. Minimum inhibitory concentration (MIC) studies of lemon grass oil against* Aspergillum fumigatum*.

## Data Availability

Not applicable.
