# Supplementary material for: Essential oil from Cymbopogon citratus exhibits “anti-aspergillosis” potential: in-silico molecular docking and in vitro studies
Source: Bull Natl Res Cent. 2022 Jan 29;46(1):23. doi: 10.1186/s42269-022-00711-5 (PMC8800409; doi:10.1186/s42269-022-00711-5)
Supplement: Supplementary file 1 — Additional file 1: Supplementary Figure 1. Minimum inhibitory concentration (MIC) studies of lemon grass oil against Aspergillum fumigatum. [file 42269_2022_711_MOESM1_ESM.docx]

50 µl

100 µl

35 µl

25 µl

15 µl

5 µl


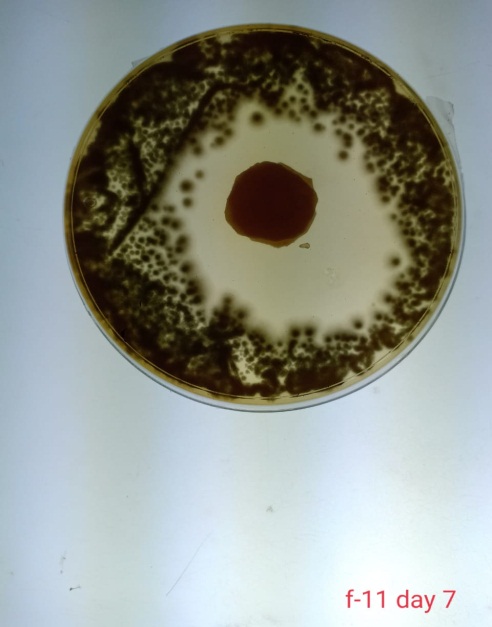

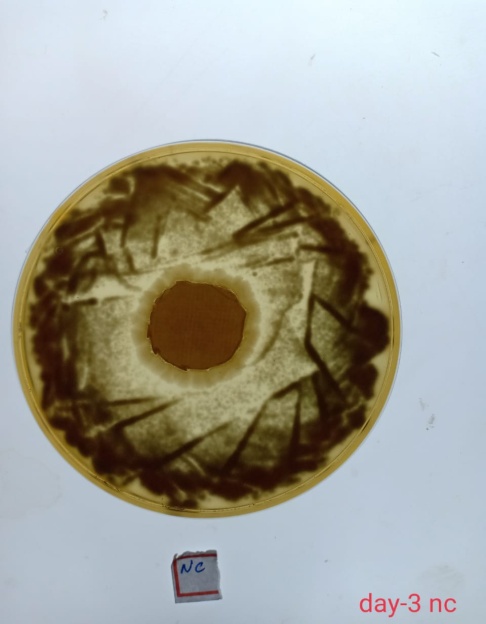

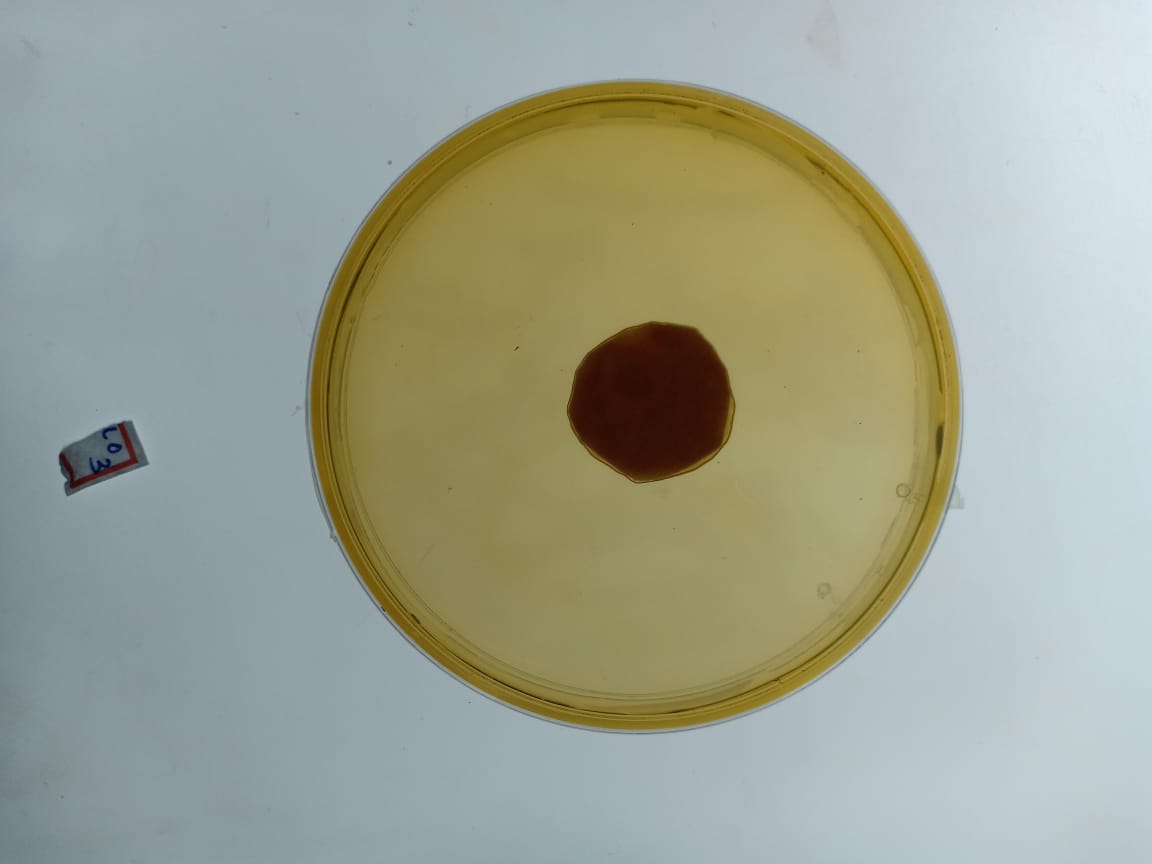

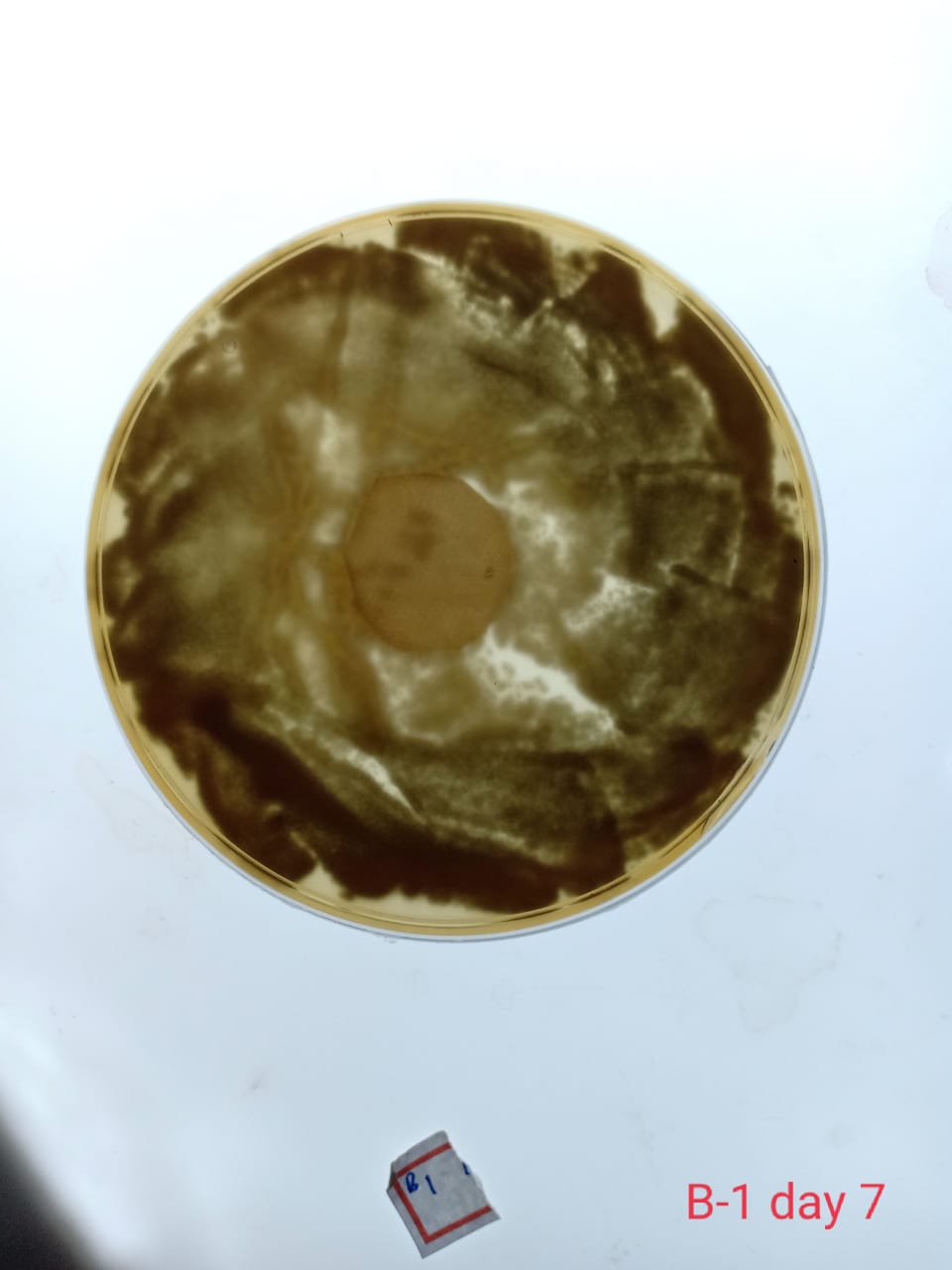

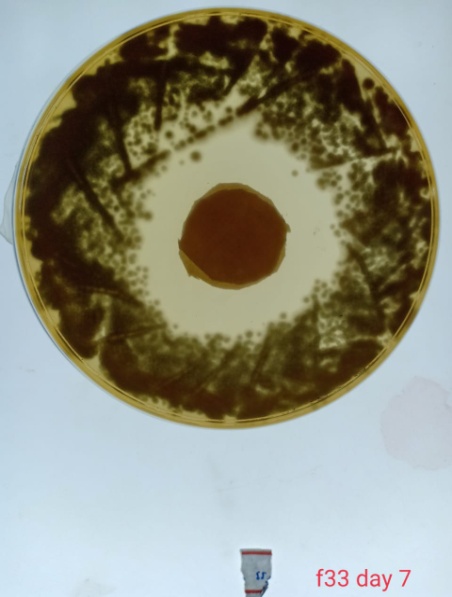

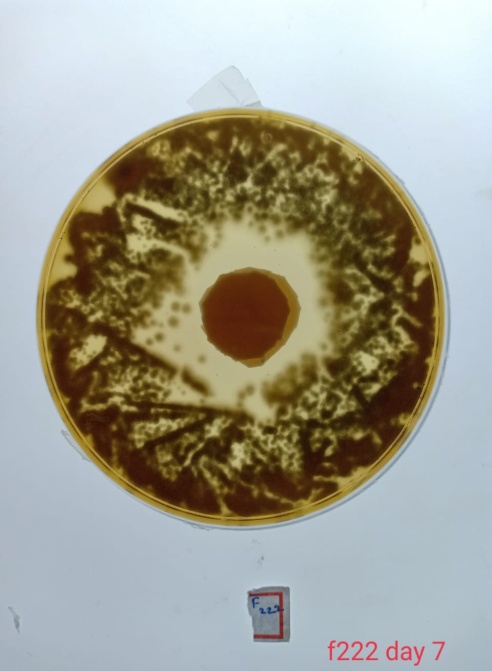


Supplementary Figure 1: Minimum inhibitory concentration (MIC) studies of lemon grass oil against *Aspergillum fumigatum*.
